# Mesenchymal Stem Cell-Derived Exosome Therapy of Microbial Diseases: From Bench to Bed

**DOI:** 10.3389/fmicb.2021.804813

**Published:** 2022-01-03

**Authors:** Xiaolan Wu, Shanshan Jin, Chengye Ding, Yu Wang, Danqing He, Yan Liu

**Affiliations:** Laboratory of Biomimetic Nanomaterials, Department of Orthodontics, Peking University School and Hospital of Stomatology and National Center of Stomatology and National Clinical Research Center for Oral Diseases and National Engineering Laboratory for Digital and Material Technology of Stomatology and Beijing Key Laboratory of Digital Stomatology and Research Center of Engineering and Technology for Computerized Dentistry Ministry of Health and NMPA Key Laboratory for Dental Materials, Beijing, China

**Keywords:** microbial diseases, exosomes, mesenchymal stem cells, cell-free therapy, antibiotic resistance

## Abstract

Microbial diseases are a global health threat, leading to tremendous casualties and economic losses. The strategy to treat microbial diseases falls into two broad categories: pathogen-directed therapy (PDT) and host-directed therapy (HDT). As the typical PDT, antibiotics or antiviral drugs directly attack bacteria or viruses through discerning specific molecules. However, drug abuse could result in antimicrobial resistance and increase infectious disease morbidity. Recently, the exosome therapy, as a HDT, has attracted extensive attentions for its potential in limiting infectious complications and targeted drug delivery. Mesenchymal stem cell-derived exosomes (MSC-Exos) are the most broadly investigated. In this review, we mainly focus on the development and recent advances of the application of MSC-Exos on microbial diseases. The review starts with the difficulties and current strategies in antimicrobial treatments, followed by a comprehensive overview of exosomes in aspect of isolation, identification, contents, and applications. Then, the underlying mechanisms of the MSC-Exo therapy in microbial diseases are discussed in depth, mainly including immunomodulation, repression of excessive inflammation, and promotion of tissue regeneration. In addition, we highlight the latest progress in the clinical translation of the MSC-Exo therapy, by summarizing related clinical trials, routes of administration, and exosome modifications. This review will provide fundamental insights and future perspectives on MSC-Exo therapy in microbial diseases from bench to bedside.

## Introduction

Microbial diseases, known as infectious diseases, refer to the clinical manifestation of damage that results from a host-microbe interaction (Casadevall and Pirofski, 2000). Infections can be classified into four broad categories based on phylogenetic groupings of microbes, bacteria, viruses, parasites, and fungi. In Global Risks Reports 2021 from World Economic Forum, infectious diseases rank first by impact among top global risks, and are a leading cause of morbidity and mortality worldwide. The predicament in microbial disease treatments is a consequence of three simultaneous factors. Firstly, antibiotic resistance presents an acute threat to the effectiveness of available antimicrobial therapies. Original pathogenic microorganisms occasionally reappear in drug-resistant forms, as exemplified by multidrug-resistant *Mycobacterium tuberculosis* (Laxminarayan et al., 2020). Secondly, the eradication of microbes is not equivalent to the termination of clinical symptoms, since immunological damages to the host may persist following a successful antipathogen response (Kaufmann et al., 2018). Finally, new pathogenic microbes keep emerging, for which no therapy exists. Since the outbreak of coronavirus disease 2019 (COVID-19) in late December 2019, it has brought tremendous casualties and economic losses to over 200 countries and regions. Although the pathogenesis of COVID-19 has been fully elucidated, there is no specific therapy for the disease at present (Tsang et al., 2021).

The invention of antimicrobial agents is a remarkable victory in the pathogen-directed therapy (PDT) to treat infectious diseases. However, the efficacy of existing antimicrobials is losing sustainability, as antimicrobials constantly pose selective pressure on mutations in the genes of drug targets (Holmes et al., 2016). It is important to come up with a novel therapy that does not exacerbate antimicrobial resistance. The host-directed therapy (HDT) is a choice, which functions by regulating host cell factors to negatively influence survival or proliferation of microorganisms (Zumla et al., 2016). The application of mesenchymal stem cell-derived exosomes (MSC-Exos) in treatments of microbial diseases is an explorative and promising HDT. Exosomes are double lipid layer vesicles ranging from 30 to 150 nm, basically composed of lipids, proteins, and nucleic acids (Doyle and Wang, 2019). MSC-Exos are commonly used as a source of acellular therapy due to their immunomodulatory, pro-reparative, and drug delivery properties. They have been studied deeply for the application in treatments of several kinds of diseases, such as neurodegenerative diseases (Guy and Offen, 2020), cancers (Vakhshiteh et al., 2019), and injuries in heart (Suzuki et al., 2017; Babaei and Rezaie, 2021), kidney (Nargesi et al., 2017), and nerve (Zhang et al., 2021b). Exosome-based cell-free vaccines are also in development against HIV-1 associated diseases (Rezaie et al., 2021) and cancers (Nikfarjam et al., 2020). Modification of exosomes *via* pre-loading or post-loading approaches can further boost the therapeutic efficacy (Madrigal et al., 2014).

In this review, we mainly focus on the application of MSC-Exos in microbial diseases. Specifically, we update current understandings of MSC-Exo therapy in periodontitis, pneumonia, sepsis, and diabetic foot ulcer (DFU) infection. Then, progresses in clinical translation of exosome therapy are summarized, with the discussion in routes of administration and exosome modification to enhance therapeutic effects of MSC-Exos. Finally, we give perspectives in the future direction of MSC-Exo therapy.

## Microbial Disease Therapeutics

Based on the target difference, microbial disease therapeutics can be categorized into two strategies: PDT and HDT (Nisini et al., 2020). In this section, we make a detailed introduction to methods involved in these two strategies, and discuss how they complement each other to improve outcomes of microbial diseases. Thereinto, MSC-Exo therapy, originating from MSC therapy, is brought up as a promising HDT candidate.

### Pathogen-Directed Therapy

Pathogen-directed therapy, as the name suggests, interacts directly with pathogens (bacteria, viruses, fungi, and parasites) to interrupt their intrusion, survival, and proliferation (Shang et al., 2020). Anti-infective drugs, as the representative, combine directly with the components of pathogens, causing death of pathogens or inhibiting their replication. Among antibacterial, antiviral, antifungal and antiparasitic drugs, antibacterial drugs are by far the most used. They function by interrupting essential bacterial activities, such as destructing cell wall integrity, depolarizing cell membrane potential, suspending DNA replication, and inhibiting protein synthesis (Kohanski et al., 2010; Leekha et al., 2011).

Although the use of antibiotics saved billions of lives in the past over half a century, shortcomings have gradually surfaced (Clardy et al., 2009). Firstly, nonstandard medication accelerates the progress of antimicrobial resistance. Broad-spectrum antibiotics abuse in common infections, which are indications for narrow-spectrum antibacterial agents, raises concerns about their effectiveness in the long term (Holmes et al., 2016). Genes encoding antibiotic resistance are continuously evolving, and are distributed to numerous bacterial species in a plasmid-mediated way (Laxminarayan et al., 2020). Protective mechanisms against antimicrobial agents include expressing drug efflux systems, modifying drug target sites, producing enzymes to destroy drugs, or producing an alternative metabolic pathway to bypass the action of the antimicrobials (Tenover, 2006). The emergence of multidrug-resistant bacteria become increasingly prevalent, such as Methicillin-resistant *Staphylococcus aureus* and vancomycin-resistant *Enterococcus*, which are the most common antibiotic-resistant bacteria (Vivas et al., 2019). Secondly, physicochemical properties of current antimicrobials hold back their efficacy. Hydrophilic antibiotics are inactive against intracellular pathogens, with narrow bio-distribution limited at the extracellular space. They show low permeability toward biological barriers, thus hard to achieve minimum inhibitory concentration at specific sites (e.g., ocular fluid, cerebrospinal fluid, and abscess cavity; Pea et al., 2005).

Other pathogen-directed strategies mainly include antimicrobial peptides (AMPs), antibodies, antimicrobial nanoparticles (NPs), and the CRISPR-Cas system. AMPs are small effector molecules produced naturally or synthetically (Tan et al., 2021), such as cathelicidins, defensins, and hepcidin (Alcayaga-Miranda et al., 2017). They exhibit direct bactericidal properties by physically destroying microbial lipid bilayers to release cell contents (Zhang et al., 2021a). Antimicrobial NPs (metal NPs, semi-conductive NPs, and organic NPs) are promising antimicrobial agents, the underlying basic mechanism of which is related to reactive oxygen species-induced interruption of bacteria membranes (Reshma et al., 2017; Calabrese et al., 2022). Antimicrobial NPs can be applied in the coating for implantable devices (Wang et al., 2017; Fernando et al., 2018) and treatments of superficial infections (Paladini and Pollini, 2019), whereas the potential toxicity of NPs should not be neglected (Xu et al., 2020). Targeting antimicrobial-resistant plasmid or bacteria genome, the CRISPR-Cas system can induce DNA damage to program bacterial death (Bikard and Barrangou, 2017; Vila, 2018). Nevertheless, the major obstacle lies in the development of specific and efficient delivery approach (Fagen et al., 2017; Fage et al., 2021). It is obvious that new remedies should be exploited to compensate drawbacks of PDT.

### Host-Directed Therapy

There is no doubt that antimicrobial therapy is the mainstream treatment for most infectious diseases. However, when confronted with complicated situations such as drug-resistant microbes, biofilm-associated infections, existing antimicrobials lose their efficacy. To counteract the emergence of antimicrobial resistance, a novel anti-infectious therapy focused on the modulation of host response (i.e., HDT) has been proposed. HDT is aimed at improving innate or adaptive protective immune response to control pathogens and/or limit immunopathology. Conventional HDT includes the application of immunomodulators, therapeutic vaccines, repurposed drugs, micronutrients, and stem cell therapy (Zumla and Maeurer, 2016).

Immunomodulatory drugs play important roles in HDT, as they not only promote protective immune responses in acute phase, but also attenuate constant, excessive inflammation in chronic stage (Kilinc et al., 2021). For example, NSAIDs are administrated in the treatment of late-stage multidrug-resistant tuberculosis, to promote phagocytosis and bacterial killing by inhibiting the production of prostaglandin E2 (PGE2) in macrophages (Kroesen et al., 2017). Therapeutic vaccines refer to the injection of pathogen antigens (proteins, nucleic acids) into patients with persistent, recurrent, or chronic infectious diseases. They aim at reducing the severity of the disease or preventing complications, by stimulating the immune defense response (Moingeon et al., 2003; Autran et al., 2004). Drug repurposing is a strategy for identifying new uses of existing drugs for non-communicable diseases (Pushpakom et al., 2019). Cholesterol-lowering drugs, asthma drugs, diabetes drugs, and anticonvulsants are common candidates (Zumla and Maeurer, 2016). Repurposed drugs outrun new drug development in terms of efficiency, lower costs, and safety. Supplementing micronutrients, such as vitamin D, zinc, and probiotics, helps build up immunity (Zumla et al., 2016). Thereinto, probiotics are a novel HDT, in which adequate amounts of probiotic bacteria or bacterial products are administrated to confer health benefits to the host. Underlying mechanisms include competitive colonization with pathogens, promotion of beneficial immune modulation, and suppression of excessive inflammation (Chibbar and Dieleman, 2015).

Stem cell therapy stands out among other host-directed therapies for its unique capability in multi-lineage differentiation and immunomodulation. Its applications in the treatment of various kinds of bacteria and virus infections are supported by solid experimental researches, meanwhile clinical trials are going through to further validate its safety and efficacy (Al-Anazi and Al-Jasser, 2015; Marrazzo et al., 2019; Sleem and Saleh, 2020). Recently, the attention on mesenchymal stem cell (MSC) therapy rockets, with more than 50 clinical trials in progress to evaluate its application on COVID-19-associated acute respiratory distress syndrome (ARDS)/pneumonia (Meng et al., 2020; Shetty et al., 2020). Other hot research fields include septic shock (McIntyre et al., 2018; Schlosser et al., 2019; Laroye et al., 2020), human immunodeficiency virus infection (Allam et al., 2013; Zhang et al., 2013), influenza-associated pneumonia (Darwish et al., 2013), hepatitis B virus-induced liver failure/cirrhosis (Peng et al., 2011; Kantarcioglu et al., 2015; Lin et al., 2017), mycobacterium tuberculosis-induced bone defects (Zhang et al., 2021c), and refractory cytomegalovirus infection (Meisel et al., 2011). MSC therapy has huge potential in adjuvant anti-infectious treatments *via* immunomodulation and tissue repair.

Early studies mainly attribute the therapeutic effects to the homing and differentiation ability of MSCs (Li et al., 2020c). However, recent researches have revealed that MSCs had short survival time after transplantation and only a small proportion of MSCs succeeded in arriving at injured sites (Burst et al., 2010; Xu et al., 2016). It is demonstrated that the essential of therapeutic effects might lie in the secretome of MSCs, which exerts immunomodulatory and reparative properties (Xie et al., 2021). MSCs have active paracrine actions, releasing large amounts of growth factors, cytokines, immunomodulators, and extracellular vesicles (EVs). EVs that are classified into apoptotic vesicles, microvesicles (MVs), and exosomes, play an important role in intercellular and even interorganismal communications. Compared to apoptotic vesicles, MVs and exosomes are the more widely investigated. Assembled in composition and functions, major differences between MVs and exosomes lie in the biogenesis pathway and size. MVs are plasma membrane-derived relatively large EVs, ranging from 100 to 1,000 nm; while exosomes are endosome-origin small EVs with a diameter of 30–150 nm (Cocucci and Meldolesi, 2015; Thery et al., 2018). Due to high similarities in constituent and limitations in available purification methods, some reports have interchangeably used the terms “exosomes” and “MVs” (Lee et al., 2012). In this review, we mainly focus on MSC-Exos, but studies on MSC-EVs or MSC-MVs are also included in consideration of comprehensiveness. Proteomic (Pierce and Kurata, 2021), metabolic, lipidomic (Showalter et al., 2019), and miRNA-sequence analysis (Shao et al., 2017; Zhao et al., 2019a) and experimental studies have indicated that MSC-Exos inherit similar biological properties from their parent cells, in aspect of immunomodulation (Willis et al., 2018), tissue repair promotion (Shao et al., 2017) and homing capacity (Shao et al., 2017; Guo et al., 2019), which are important properties for treatments of microbial diseases. What is more, exosome therapy is superior to stem cell therapy in biosafety. Reports about adverse events of MSC therapy are not uncommon and concerns about MSC therapy have never ceased. There are worries about tumorigenesis, disease transmission, undesired immune responses, replantation on unwanted sites, and administration site reactions (Prockop et al., 2010; Barkholt et al., 2013; Casiraghi et al., 2013; Arango-Rodriguez et al., 2015). In contrast, few serious adverse events are reported in MSC-Exo therapy. Taken comparable biological properties and superior biosafety, MSC-Exo therapy might be a better choice for HDT.

## Mesenchymal Stem Cell-Derived Exosomes

As described in the previous section, MSC-Exos have received increasing attentions for therapeutic administration. Furthermore, a variety of clinical trials are underway for the application of MSC-Exos as a novel, safe and efficacious cell-free therapy in microbial diseases. Below, we give a global perspective regarding biogenesis, isolation, and characterization, as well as their molecular composition of exosomes.

### Biogenesis of Exosomes

**Figure 1:**
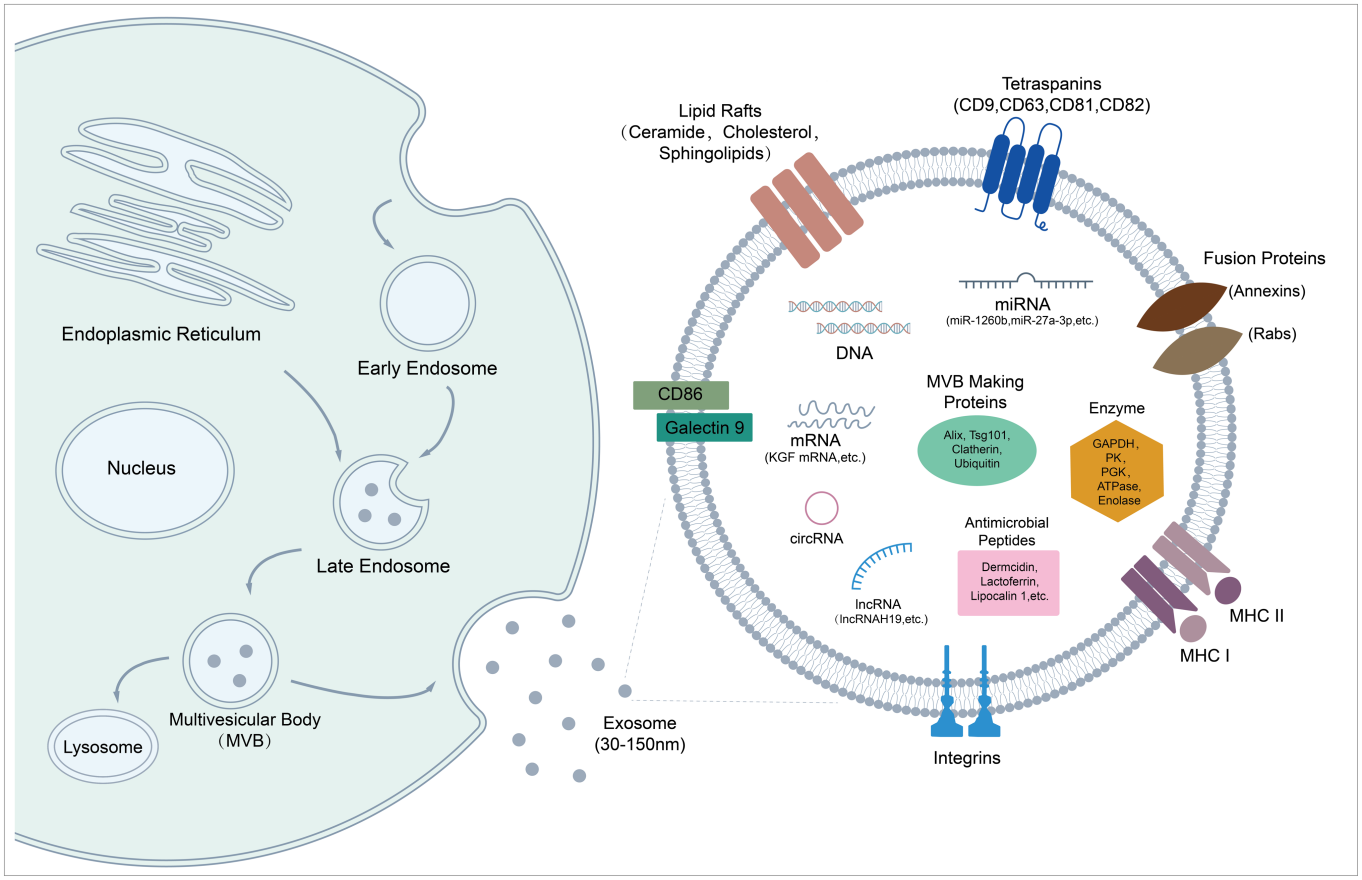
Biogenesis and molecular composition of exosomes. Schematic diagram shows exosome formation and biological cargoes. Exosomes originate in endosome system, and are released from cells as particles (30–150 nm) with a lipid bilayer. They are endowed with therapeutic potential by carrying various kinds of bioactive molecules, such as peptides, microRNAs, and mRNAs.

Exosomes originate in the endosome system, and this process is mediated by several molecules, as illustrated in Figure 1. Early endosomes are formed by invagination of the plasma membrane during endocytosis (Huotari and Helenius, 2011). Then they mature into late endosomes, which turn into multivesicular bodies (MVBs), when exosomes are generated as intraluminal vesicles (ILVs) by invagination of late endosome membrane. The MVBs can either be degraded by lysosomes or released into extracellular matrix as exosomes *via* exocytosis (Kowal et al., 2014; Hessvik and Llorente, 2018; Zhang et al., 2019). It has been established that exosomes are actively secreted by almost all cell types especially MSCs, as we describe in detail later. MSCs can be derived from different tissues, such as bone marrow, adipose tissue, dental pulp, and menstrual blood (Shi et al., 2021b). The secreted exosomes could be taken up by recipient cells *via* endocytosis, phagocytosis, or direct membrane fusion, then the contained bioactive cargos are transferred to modify gene expression, signaling, and overall functions and behaviors of recipient cells (Fujita et al., 2015; Rani and Ritter, 2016).

### Isolation and Characterization of Exosomes

Exosomes can be directly isolated from cell culture medium or biological fluids, such as urine, breast milk, and amniotic fluid (Thery et al., 2018). The isolation strategies include ultracentrifugation, polymer precipitation, size-exclusion chromatography, and immunoaffinity capture. Ultracentrifugation is the most commonly-used isolation method in basic researches (Gardiner et al., 2016). The typical ultracentrifugation protocol includes: (i) low-speed (300 × *g* and 2,000 × *g*) centrifugation to remove cells and dead cells respectively; (ii) higher-speed centrifugation (10,000 × *g*) to remove cell debris; and (iii) high-speed centrifugation (100,000 × *g*) to pellet exosomes (Thery et al., 2006). Time consumption and complex operation procedures remain the main disadvantages of ultracentrifugation. Isolation with proprietary polymer precipitation and centrifugation is convenient for small volume samples, but the biggest problem lies in the low purity of exosomes with the contamination of miscellaneous proteins and polymers (Coughlan et al., 2020). Size-exclusion chromatography can screen exosomes of high purity and integrity, in which molecules filtrate through gels at different speeds depending on size difference (Sidhom et al., 2020). In addition, immunoaffinity capture can recover exosomes from complex and viscous fluids, making it a good choice for clinical diagnosis with small-volume plasm (Yang et al., 2017).

Followed by isolation, characterization of exosomes is necessary before therapeutic administration and mechanistic explanation. Various techniques have been developed to confirm the biochemical, biophysical, and biomechanical properties of exosomes. Western blotting is the main tool for general biochemical characterization. MISEV2018 guidelines require the identification of at least three positive protein markers of EVs: (i) transmembrane proteins or GPI-anchored proteins (e.g., CD63, CD81, and CD9); (ii) cytosolic proteins recovered in EVs (e.g., TSG101, ANXA, and HSPA8); and (iii) major components of non-EV co-isolated structures for purity control (e.g., albumin and ribosomal proteins; Thery et al., 2018). For biophysical characterization of single vesicles, electron or atomic force microscopy is necessary to provide both close-up and wide-field images. Apart from that, other techniques are available as a supplement to estimate size, light scattering, and fluorescence properties of exosomes. Tunable resistive pulse sensing provides reliable and fast particle-by-particle measurement of EV size and concentration distribution (Vogel et al., 2016). Nanoparticle tracking analysis can visualize and track the Brownian motion of individual vesicles by light scattering, and make calculation of size distribution and total concentration (Sokolova et al., 2011). High resolution flow cytometry is applicable for exosome immunophenotyping (Nolan and Duggan, 2018).

### Molecular Composition of Exosomes

Exosomes are vesicles with a diameter of 30–150 nm, mainly composed of lipids, proteins, and nucleic acids (Figure 1; Doyle and Wang, 2019). Exosomes inherit similar but different substances and biological properties from their parent cells. Compared to parent cells, the double membrane structure of exosomes contains a higher content of unsaturated phospholipids and a higher ratio of lipid/protein, which increases the rigidity of exosomes, ensuring relative stability of exosomes in biologic fluids. Furthermore, integrin-associated proteins on the surface protect vesicles from phagocytosis of mononuclear phagocytic system (MPS) to certain extent (Record, 2018). Currently, nucleic acids and proteins are considered as main participants of exosome treatments (Tan et al., 2015). MSC-Exos are enriched in miRNAs with different functions, such as anti-inflammatory miRNAs, anti-apoptotic miRNAs, and immunoregulatory miRNAs (Schultz et al., 2021). Some studies report therapeutic roles of exosomal mRNA and other non-coding RNA (lncRNA, cirRNA, and piRNA) in microbial diseases (Zhu et al., 2014; Li et al., 2020a; Shi et al., 2020; Yu et al., 2020a). In addition, protein profiling of MSC-EVs reveals that exosomal proteins are related to biological process such as innate immunity, antimicrobial, host-virus interaction, cellular oxidant detoxification, and complement and coagulation cascades. Several AMPs were identified, including dermcidin, lactoferrin, lipocalin 1, lysozyme C, neutrophil defensin 1, S100A7 (psoriasin), S100A8/A9 (calprotectin), and histone H4 (Pierce and Kurata, 2021). AMPs partially account for the antimicrobial effects of MSCs’ secretome, which may also work in terms of MSC-Exos (Alcayaga-Miranda et al., 2017).

## MSC-Exos for Therapeutic Applications in Microbial Diseases

**Figure 2:**
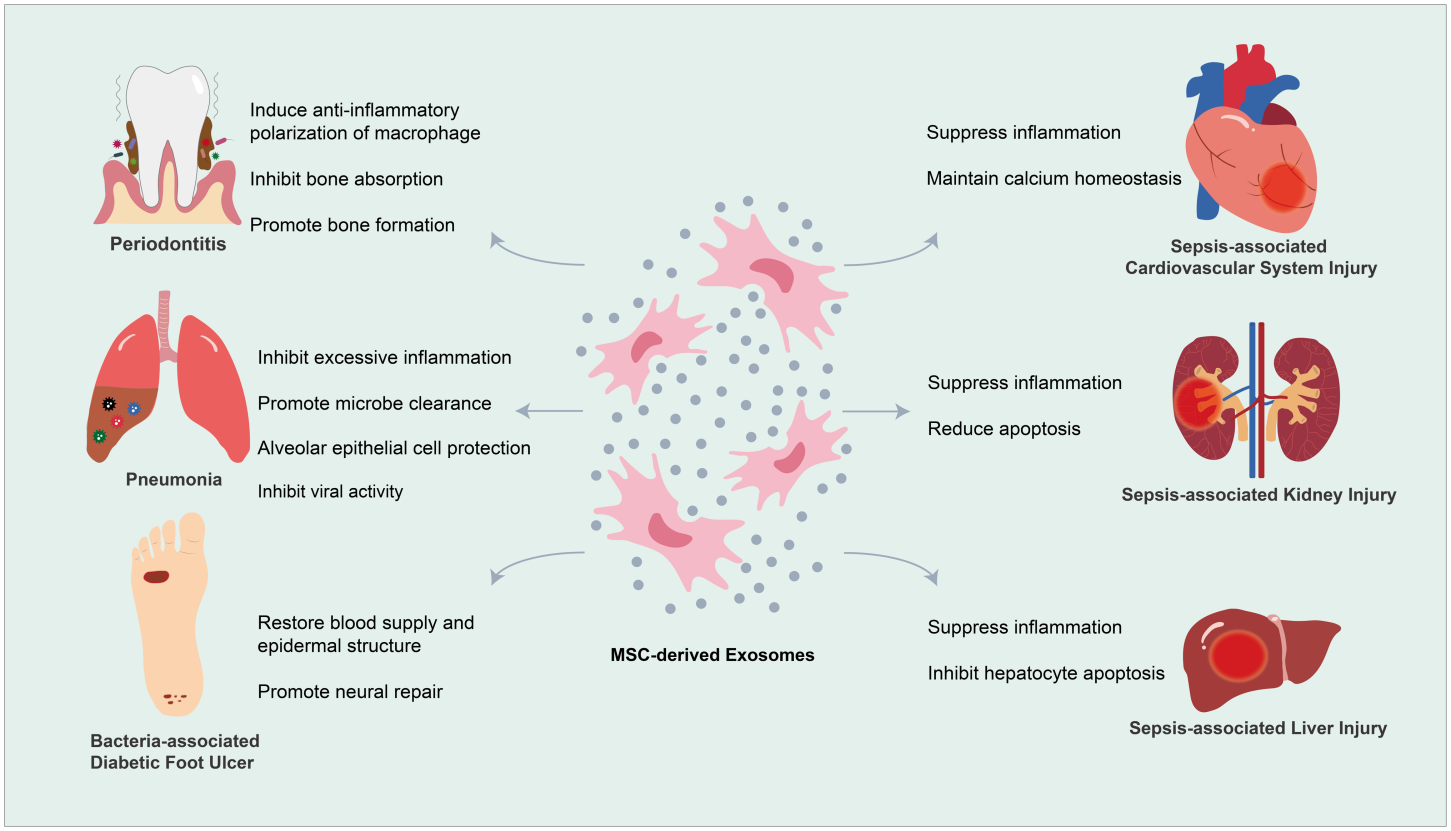
Mesenchymal stem cell-derived exosomes (MSC-Exos) for therapeutic application in microbial diseases.

The idea of bringing MSC-Exos into HDT for infectious diseases is explorative. In this section, we mainly focus on summarizing experimental proves for the efficacy of MSC-Exos in the treatment of some persistent or refractory infectious diseases. We first start with a topical disease, periodontitis, and then discuss multiple systematic diseases, including bacteria/viruses-associated pneumonia, sepsis, and bacteria-associated DFUs (Figure 2).

### MSC-Exo Therapy for Periodontitis

Periodontitis refers to the inflammatory destruction of the periodontal supportive tissue (gingiva, periodontal ligament, and alveolar bone) as a result of polymicrobial colonization on tooth surfaces in the form of biofilms. Periodontitis has been recognized and treated for at least 5,000 years, and the classification of which has been changed and evolved with the development of new knowledge. Several microbes are associated with specific types of periodontal diseases, such as *Aggregatibacter actinomycetemcomitans* with aggressive periodontitis, and *Porphyromonas gingivalis* with severe or progressive periodontitis. The presence of the microbial biofilm might not be sufficient to directly cause periodontal disease. Periodontitis occurs when the balance between microbial biofilms and immune responses of the host is lost (Kinane et al., 2007; Kinane and Hajishengallis, 2009; Hajishengallis and Lamont, 2012). As pathogens invade periodontium, immune cells release anti-inflammatory cytokines and antibacterial molecules to fight against pathogens, upsetting the homeostasis of alveolar bone at the same time. Mechanistically, a cascade of events activates osteoclastogenesis leading to subsequent alveolar bone loss *via* the receptor activator of nuclear factor-kappa B (RANK)- ligand (RANKL)-osteoprotegerin (OPG) axis (Cochran, 2008; Barbato et al., 2015). Moreover, periodontitis is a disease of high morbidity and recurrence (Frencken et al., 2017). Progressive alveolar bone loss ultimately leads to loss of teeth, posing negative influences to oral function and aesthetics. The harm of periodontitis exceeds teeth loss, but also involves a higher risk of systematic diseases, such as cardiovascular disease (Donders et al., 2021), oral squamous cell carcinoma (Hu et al., 2021), and rheumatoid arthritis (Choi and Lee, 2021).

Routine treatments for periodontal diseases include mechanical approaches, scaling and root planning, to remove microbial biofilms. *In situ* or systematic antibiotics are applied as adjunctive therapies when periodontal infection is hard to control. Yet, the recurrence of periodontal disease is high, and no mechanical techniques rescue the loss of alveolar bone (Kinane et al., 2017). MSC-Exo therapy stands out in the treatment of periodontitis for its ability in suppressing excess inflammation and promoting tissue regeneration simultaneously. In treatment of periodontitis, MSC-Exos, often co-assembled with tissue engineering scaffolds, are implanted into periodontal bone defects to promote the regeneration of periodontal supportive tissues. Abundant studies have demonstrated that the regulation of MSC-Exos involves several kinds of cells, such as macrophages, osteoclasts, and periodontal ligament cells in periodontium (Yu et al., 2020b; Gegout et al., 2021).

**Figure 3:**
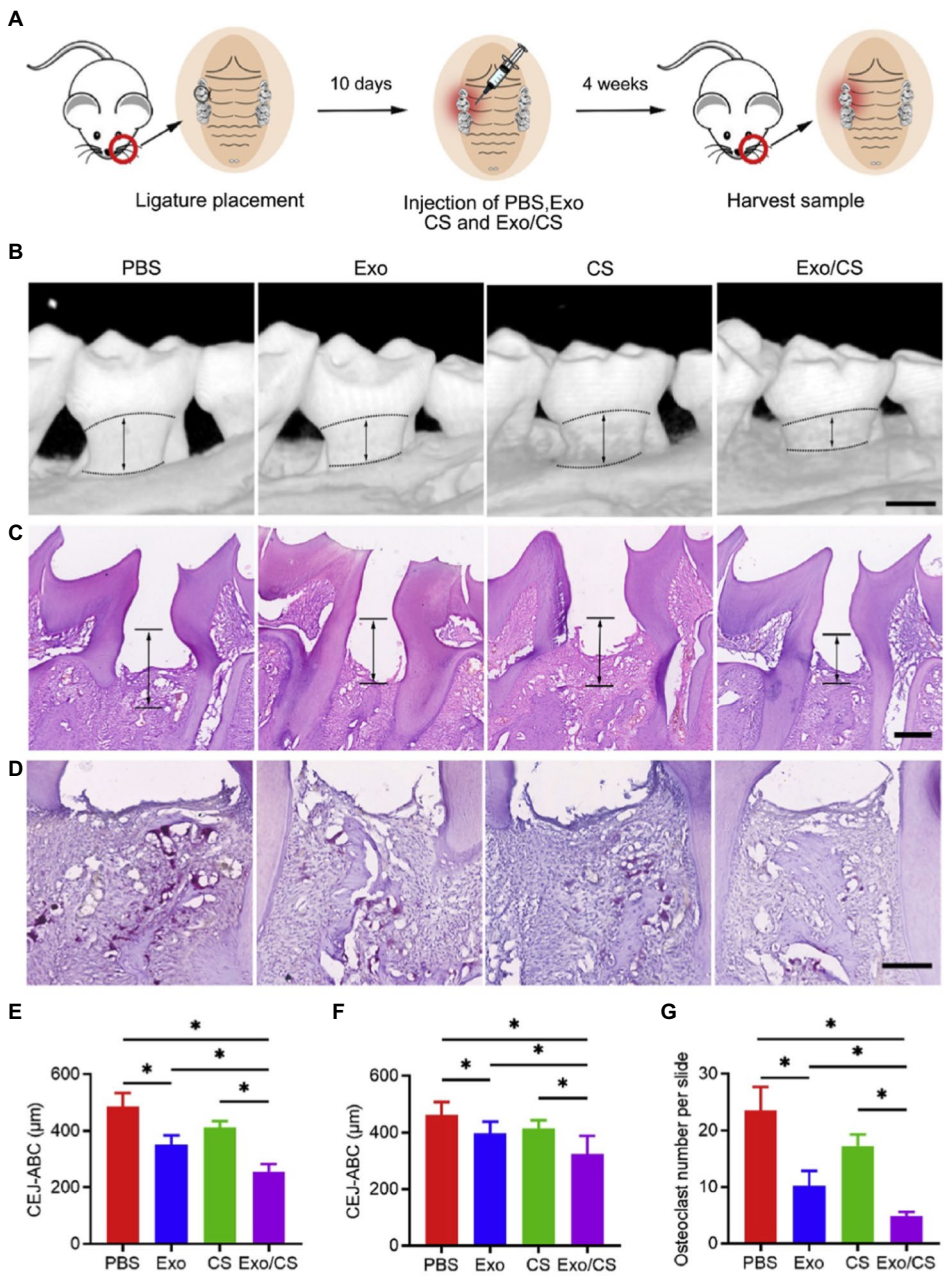
Dental pulp stem cell-derived exosomes (DPSC-Exos)-incorporated chitosan hydrogel (DPSC-Exo/CS) rescues epithelial lesion and alveolar bone loss in mice with experimental periodontitis. **(A)** Schematic illustration. **(B)** 3D micro-CT reconstructions of maxillae of PBS-, CS-, DPSC-Exo- and DPSC-Exo/CS-treated groups (*n* = 6 per group). **(C)** Histological H&E-stained sections of the periodontium from each group. **(D)** Histological tartrate-resistant acid phosphatase (TRAP)-stained sections of the periodontium from each group. The number of osteoclasts was quantified in each microscope field of view. **(E,F)** Statistical analysis of the CEJ-ABC distance in each group (*n* = 6 per group) as determined by micro-CT and H&E staining, respectively. **(G)** Statistical analysis of the number of osteoclasts in each group (*n* = 6 per group) as determined by TRAP staining. Error bar represents SEM. ^*^p < 0.05 (Adopted from Shen et al., 2020 distributed under the CC BY-NC-ND license).

Macrophages are crucial immunomodulators of the periodontal disease and account for both initiation and resolution of inflammation and osteoclastogenesis (Darveau, 2010; Hienz et al., 2015). Macrophages can be polarized into pro-inflammatory phenotype (M1 macrophage) and anti-inflammatory phenotype (M2 macrophage), to mediate inflammation and maintain tissue homeostasis, respectively (Shapouri-Moghaddam et al., 2018; Jin et al., 2019). MSC-Exo therapy inhibits excessive inflammation in periodontium by converting M1 macrophages into M2 macrophages. Shen et al. (2020) injected dental pulp stem cell-derived exosomes (DPSC-Exos) and DPSC-Exo-incorporated chitosan hydrogel (DPSC-Exos/CS) respectively into periodontal pockets of ligature-induced periodontitis mice. Both DPSC-Exos and DPSC-Exo/CS rescued alveolar bone loss and periodontal epithelial lesion to some degree, with the chitosan hydrogel one performing better (Figure 3). Mechanistically, it was demonstrated that DPSC-Exos delivered miR-1246 to induce anti-inflammatory polarization of macrophage, and downregulated NF-κB p65 and p38 mitogen-activated protein kinase (MAPK) signaling pathways to alleviate periodontal inflammation (Shen et al., 2020). In another research, Nakao et al. (2021) locally injected human gingiva-derived MSC-derived exosomes (GMSC-Exos) into periodontal pockets of mice, and observed reduced bone resorption and the number of tartrate-resistant acid phosphatase (TRAP)-positive osteoclasts in periodontal tissue, and these effects were further enhanced by pretreating GMSCs with TNF-α. Delivery of exosomal miR-1260b accounts for the anti-osteoclastogenic ability of GMSC-Exos, which targets Wnt5a-mediated RANKL pathway (Nakao et al., 2021). Analogously, decreased RANKL/OPG ratio and number of TRAP-positive cells indicate inhibition of osteoclastogenesis by bone marrow mesenchymal stem cell-derived exosomes (BMSC-Exos) in periodontitis rats (Liu et al., 2021).

In addition to the immunomodulation of MSC-Exos in the treatment of periodontitis, other studies indicate MSC-Exos rescue the osteogenic ability of stem cells in periodontal ligaments. Wei et al. (2020) indicated human exfoliated deciduous teeth (SHED-Exos) promoted BMSCs osteogenesis, differentiation, and bone formation *via* Smad5 signaling in a ligature-induced periodontitis mouse model. Consistently, Wang et al. (2020c) demonstrated the role of SHED-Exos in enhancing the osteogenic differentiation of periodontal ligament stem cells (PDLSCs) *via* Wnt and BMP signaling *in vitro*. Enhancing angiogenesis is another important strategy in promoting delayed bone healing, as vascular system supplies nutrients, oxygen, and serves as a niche for osteoprogenitor cells during bone repair (Liu and Castillo, 2018). In the study of Wu et al. (2019), SHED-Exos/β-tricalcium phosphate targeted AMPK signaling pathway to promote the coupling of human umbilical vein endothelial cells angiogenesis and BMSC osteogenesis in a rat periodontal defect model. Chew et al. (2019) reported MSC-Exo-loaded collagen sponge promoted regeneration of periodontal defects by enhancing viability, proliferation, and migration of periodontal ligament cells through CD73-mediated adenosine receptor activation of pro-survival AKT and ERK signaling pathways. The above researches indicate that the latent ability of MSC-Exos to regulate inflammation and bone remodeling paves the way for the establishment of a therapy for periodontitis.

### MSC-Exo Therapy for Pneumonia

Pneumonia, an inflammation of lung parenchyma, usually caused by infections, remains a heavy global burden on health (Watkins and Sridhar, 2018). According to Global Burden of Disease Study 2019, lower respiratory infections ranked fourth in leading causes of all ages, which pose severe health threat on people with a weak immunity system, especially children younger than 10 years and the elderly aged more than 75 years (Vos et al., 2020). Pneumonia starts with the pathogen invasion into the lower respiratory tract, which induces alveoli and interstitium inflammation, and pulmonary vascular congestion. As pulmonary permeability increases, transudate fluid and debris in the alveolar sacs compromise gas exchange (Brooks, 2020). Pneumonia can develop into ARDS and acute lung injury (ALI), the mortality rate of which is as high as 43% (Zambon and Vincent, 2008). Novel pharmacologic therapies for the treatment of ARDS/ALI including surfactant, vasodilators, prostacyclin, anti-inflammatory, and anti-oxidant reagents, have not yet proven to be effective (Cepkova and Matthay, 2006). In terms of promising new therapies, MSC-Exos have been explored in both preclinical and clinical studies. Accumulating evidence has demonstrated that MSC-Exo therapy is effective in attenuating excessive inflammation, restoring pulmonary function, and reducing mortality, verified in several typical ARDS/ALI animal models (Kannan et al., 2009; Knapp, 2009; Islam et al., 2012; Zhu et al., 2014; Hraiech et al., 2015; Monsel et al., 2015; Ogata-Suetsugu et al., 2017; Hao et al., 2019; Domscheit et al., 2020; Metcalfe, 2020; Wang et al., 2020a; Kaspi et al., 2021; Shi et al., 2021a; Tieu et al., 2021). Herein, the following portion aims at providing a comprehensive understanding of therapeutical mechanisms of MSC-Exos on bacteria/viruses-induced pneumonia.

#### Inhibiting Excessive Inflammation

Mesenchymal stem cell-derived exosomes exhibit immunomodulatory properties by directly targeting innate immune system. Innate immunity cells (monocytes, macrophages, and neutrophils) protect the host against infections by secretion of antimicrobial molecules and phagocytosis. However, excessive activated macrophages and neutrophils can damage alveolar epithelium and lung endothelium *via* secretion of proinflammatory cytokines, oxidants, and proteases. Recovery of intact epithelium and endothelium depends on the cessation of inflammatory injury (Matthay and Zemans, 2011). The anti-inflammation effect of MSC-Exos is repetitively proved in a lipopolysaccharide (LPS)-induced ALI mouse model, which is the most widely used and simplified model for ARDS/ALI, simulating the pulmonary response to bacterial endotoxin [7].

**Figure 4:**
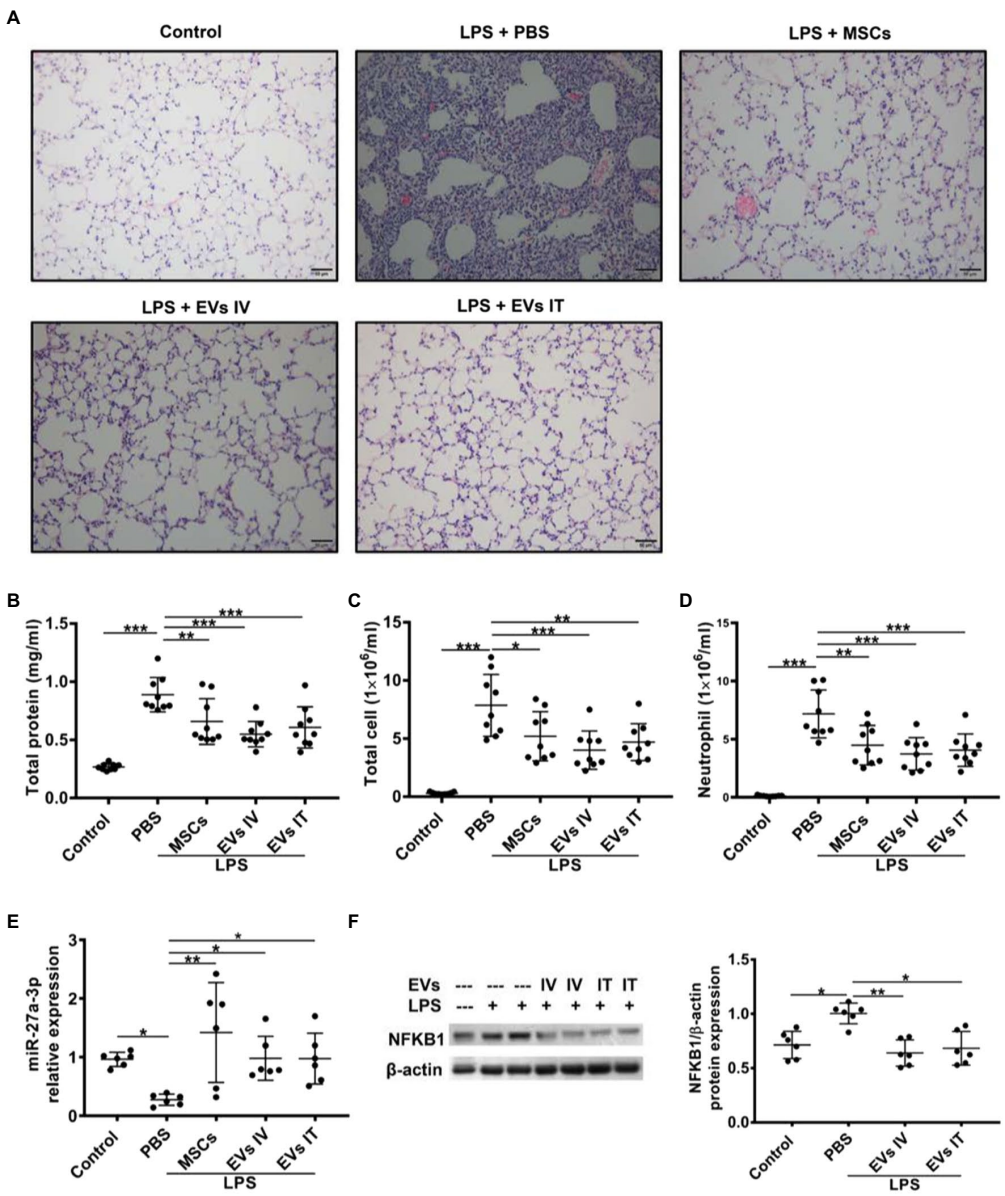
Both IV and intratracheal (IT) administration of mesenchymal stem cell-extracellular vesicles (MSC-EVs) alleviate lipopolysaccharide (LPS)-induced lung injury, elevate miR-27a-3p levels, and decrease NFKB1 levels. **(A)** Similar to the effects of MSCs, administration of EVs *via* both IV and IT dramatically improved lung injury as shown in histology. Both EVs IV and IT decreased protein concentrations **(B)**, total cell counts **(C)**, and neutrophil counts **(D)** in the bronchoalveolar lavage (BAL) harvested at 48 h after LPS insult. **(E)** Alveolar macrophages were separated 48 h after LPS insult and assayed for miR-27a-3p expression *via* quantitative real-time PCR. Results are presented relative to control group. **(F)** Alveolar macrophages were separated from BAL 48 h after IT LPS insult and assayed for NFKB1 expression *via* Western blot analysis. Data are expressed as mean ± sd; n = 6. One-way analysis of variance with Bonferroni post hoc test **(B-D)** or Kruskal-Wallis test with Dunn post hoc test **(E,F)** was used for the analysis. ^*^p < 0.05; ^**^p < 0.01; and ^***^p < 0.001. (Adopted from Wang et al., 2020a distributed under the creative commons CC BY license).

Mesenchymal stem cell-derived exosomes attenuate inflammation development and progression by regulating macrophage polarization by targeting intracellular signaling pathways or cellular metabolic pathways. In one aspect, MSC-Exos target the downstream pathway of pattern-recognition receptors (PRRs), such as NF-κB signaling pathway (Liu et al., 2017). MiR-27a-3p from MSC-EVs downregulated the expression of nuclear factor kappa B subunit 1 to promote M2 macrophage polarization, evidenced by elevated expressions of M2 markers arginase-1, interleukin (IL)-10, and decreased levels of M1 marker inducible nitric oxide synthase. Significantly reduced proinflammatory cytokines including IL-1β, IL-6, and TNF-α in the bronchoalveolar lavage (BAL) were observed (Figure 4; Wang et al., 2020a). MVs from Toll-like receptor 3 preactivated-MSCs further decreased TNF-α and increased IL-10 secretion of monocytes, which might be involved with the transfer of cyclooxygenase 2 (COX2) mRNA from MSC-MVs to monocytes. The increase in COX2, the key enzyme in PGE2 synthesis, shifted monocytes toward an anti-inflammatory phenotype by promoting PGE2 secretion (Monsel et al., 2015). In another aspect, MSC-Exos control the activation state and function of macrophages by reprogramming intracellular metabolisms. M1 and M2 macrophages exhibit different metabolic patterns. The former relies more on aerobic glycolysis, whereas the latter mainly employ mitochondrial oxidative phosphorylation (Zhu et al., 2015). BMSC-exos attenuated M1 macrophage polarization through inhibiting glycolysis, proved by decreased levels of end-products of aerobic glycolysis (adenosine triphosphate and lactic acid). Specifically, BMSC-Exos functioned by downregulating hypoxia-inducible factor 1 to inhibit the expression of rate-limiting proteins of glycolysis (Zhong et al., 2019; Deng et al., 2020). Morrison et al. (2017) reported the role of functional mitochondrial transfer through MSC-EVs in the conversion of macrophages into an anti-inflammatory phenotype *via* augmented oxidative phosphorylation (Morrison et al., 2017).

Moreover, MSC-Exos facilitates the resolution of inflammation by intermitting neutrophil migration towards lung epithelia. Hao et al. (2019) reported BMMSC-EVs reduced infiltration of white blood cells, neutrophils, and levels of TNF-α by elevating the level of extracellular leukotriene A_4_ hydrolase (LTA_4_H), proved in both *Escherichia coli* endotoxin-induced acute lung injury and *E. coli* pneumonia mouse models. LTA_4_H reduced inflammation by degrading the matrikine proline–glycine–proline, a neutrophil chemoattractant (Patel and Snelgrove, 2018). Similarly, in a *Pseudomonas aeruginosa*-induced pneumonia mouse model, nebulized human adipose-derived MSC-EVs (ADSC-EVs) reduced the inflammatory cell counts, and levels of IL-6, and TNF-α in BAL fluid. The researchers reported a dose-response effect of ADSC-EVs. Within 2 × 10^5^ to 2 × 10^6^ particles per administration, mice survival rates and EV dosage were positively correlated. However, once exceeding the dose of 2 × 10^6^ particles, ADSC-EVs posed an adverse effect on the survival rate (Shi et al., 2021a). A numerically lower influx of neutrophils was also seen in an *ex vivo* perfused human lung injured with severe *E. coli* pneumonia, after MSC-MV treatment (Park et al., 2019).

#### Promoting Microbe Clearance

Phagocytes (e.g., monocytes, macrophages, and neutrophils) safeguard lung tissue against infectious insult through the ingestion and phagocytosis of microbes (Kaufmann and Dorhoi, 2016). It has been reported that MSC-MV treatment dramatically increased bacterial phagocytosis *via* freshly isolated human alveolar macrophages (Park et al., 2019). Pro-bacterial killing effects of MSC-Exos were demonstrated in intratracheal instillation of bacteria-induced ALI mouse models, which better mimicked immune response of pneumonia patients (Knapp, 2009). In an *E. coli* pneumonia mouse model, miR-145 from BMSC-EVs decreased the activity of multidrug resistance-associated protein 1 (MRP1) in monocytes, an ATP-binding cassette transporter, to increase Leukotriene B4 production, which exerts antimicrobial effects by augmenting phagocytosis and the release of antimicrobial agents (Hao et al., 2019). Besides, Monsel et al. (2015) demonstrated increased monocyte bacterial phagocytosis after administration of MSC-MVs on *E. coli* pneumonia in mice. It is attributed to the upregulated protein level of keratinocyte growth factor (KGF) in the alveolus, which promoted bacterial clearance by decreasing apoptosis of monocytes through AKT phosphorylation (Lee et al., 2013).

#### Alveolar Epithelial Cell Protection

Mesenchymal stem cell-derived exosomes can also restore function of injured alveolar epithelial type II cells, which play an important role in the maintenance of alveolar integrity and activation of immune defense (Kannan et al., 2009). Lee et al. (2009) made deep explorations into the detailed mechanisms of epithelial cell protection effect of MSC-MVs (Zhu et al., 2014; Park et al., 2019). In an LPS-induced ALI mouse model (Zhu et al., 2014) and severe *E. coli* pneumonia *ex vivo* human lung model (Park et al., 2019), MSC-MVs dramatically improved alveolar fluid clearance and decreased lung protein permeability by the delivery of KGF mRNA to alveolar epithelial type II cells. KGF was proved effective in upregulating the key epithelial sodium channel in alveolar epithelial cells to increase fluid absorption (Lee et al., 2009). Injured alveolar epithelial type II cells benefited from the restoration of ATP levels, which might be attributed to the transfer of key metabolic enzymes (such as glyceraldehyde 3-phosphate dehydrogenase and pyruvate kinase) or mRNA for key mitochondrial genes carried by MVs in an *E. coli* pneumonia mouse model (Monsel et al., 2015). Bioenergetics reprogramming of epithelial cells can also be mediated by BMSC-EVs mitochondria transfer, reported by Islam et al. (2012).

#### Inhibiting Viral Activity

In case of viral pneumonia, apart from inhibition of cytokine storm, suppression on viral replication and attack on viruses are underlying mechanisms of MSC-Exo therapy. Exosomal microRNAs derived from MSCs might target viral genome to interfere with viral RNA transcription or protein translation essential for viral replication (Qian et al., 2016; Demirci and Adan, 2020; Sardar et al., 2020). Khatri et al. (2018) reported that MSC-EVs attenuated influenza virus-induced ALI in pigs by inhibiting viral replication, evidenced by significantly decreased virus loads in both lung lysates and nasal swabs. Meanwhile, *in vitro* experiment proved reduced virus activity in hemagglutination, replication, and pro-apoptosis. The anti-influenza property is attributed to the transfer of exosomal RNAs to epithelial cells, as therapeutic effects were reversed by pre-incubation of MSC-EVs with RNase enzyme (Khatri et al., 2018). It is presumable that miRNAs might prevent viral replication by targeting viral genes (e.g., reducing the spike protein) or inhibiting the expression of host cells receptors to avoid virus-cell interaction (Chauhan et al., 2021). Similar inhibition in viral replication was observed in the application of exosomes/microvesicles derived from murine hypothalamic neural stem/progenitor cells (htNSC) on pseudotyped SRAS-CoV-2-infected human respiratory cells *in vitro*. Furthermore, NSC-Exos exerted inherent antiviral ability by attacking and degrading viruses independent of cells. TEM imaging confirmed direct exosome-virus interaction in a cell-free environment, suggesting exosomes functioned by surrounding, engulfing, and breaking down viruses. Pretreated viruses with NSC-Exos led to degradation of spike glycoprotein and lessened infection ability toward cells (Yu et al., 2020a). However, the underlying molecular mechanisms remain to be explored.

Taken together, MSC-Exo therapy has great potential in treatment of infectious pneumonia with the ability to modulate protective immune response, provide epithelial cell protection, and inhibit viral-cell inhibition. Multiple relevant clinical trials are in progress, which will be discussed in the Section “Clinical Translation of Exosome Therapy.”

### MSC-Exo Therapy for Sepsis

Sepsis is defined as life-threatening organ dysfunction caused by a dysregulated host response to infections, according to the third international consensus definition (Singer et al., 2016). Sepsis-related death accounts for 19.7% of all global death in 2017, remaining a major public health problem (Rudd et al., 2020). Common causative microorganisms include Gram-positive bacterial pathogens (e.g., Staphylococcus aureus, *Streptococcus pneumoniae*), and Gram-negative pathogens (e.g., *E. coli*, *Klebsiella* spp., and *P. aeruginosa*; Opal et al., 2003; Umemura et al., 2021). The pathophysiologic mechanisms of sepsis can be generally concluded into three aspects: inflammation, microcirculatory dysfunction, and metabolic reprogramming (Peerapornratana et al., 2019). The possibility of polymicrobial infections adds difficulties to the early-stage diagnosis of sepsis. Empiric combined broad-spectrum antimicrobial therapy is recommended at the initial stage (Rhodes et al., 2017). However, it acts as a double-edged sword, with side effects such as increased risks of multidrug-resistant infections, organ damages, and anaphylaxis (Klompas et al., 2018; De Waele and Dhaese, 2019). In absence of such side effects, MSC-Exos may complement as an adjuvant therapy in sepsis, with their indistinctive host protection against a broad range of microorganisms and reparative effects on injured organs. MSC-Exo therapy in remedy of sepsis-induced acute injuries in liver, kidney, and cardiovascular system will be discussed in detail as follows.

#### Liver

The incidence of liver failure is relatively low in sepsis because of its ability in clearance of endotoxins and self-regeneration (Weiss et al., 2001). However, once intestinal barrier compromised under sustained systematic inflammation, bacterial translocation from the gut lumen through circulation can result in severe liver dysfunction (Sun et al., 2020). MSC-Exos show hepatic protection in condition of acute liver injury, demonstrated by improved hepatic function indicators (lower serum alanine aminotransferase and aspartate aminotransferase levels), histological characteristics changes (lower degree of hepatocellular necrosis and inflammation), and survival rates in D-GalN/LPS-induced acute liver injury mouse models (Lou et al., 2017; Hu et al., 2020; Zhang et al., 2020b). In one aspect, therapeutic effects of MSC-Exos lie in the modulation of innate immune system. Chen et al. (2017) reported exosomes from human menstrual blood-derived stem cells (MenSC-Exos) inhibited recruitment of NK cells, macrophages and release of inflammatory cytokines including TNF-α, IL-6, and IL-1β in liver. Liu et al. (2018) reported miR-17-containing ADSC-Exos suppressed the activation of thioredoxin-interacting protein-mediated NLRP3 inflammasome in hepatic macrophages, indicated by reduced cleaved-Caspase-1, IL-1β, and IL-18 expressions. Similar inhibition effects on the activity of the NLRP3 inflammasome by human umbilical cord mesenchymal stem cell (hucMSC)-Exos were observed (Jiang et al., 2019). Furthermore, Shao et al. (2020) confirmed that exosomes from IL-6 preconditioned-hucMSCs targeted phosphatidylinositol-3-kinase (PI3K) signaling pathways to suppress monocyte/macrophage activation and inflammatory cytokine secretion by transfer of miR-455-3p. In another aspect, MSC-Exos participate in the maintenance of hepatocyte hemostasis by inhibiting cell apoptosis. MenSC-Exos suppressed apoptosis by downregulation of Caspase-3, an important apoptosis-associated protein (Chen et al., 2017). Zhao et al. (2019b) reported BMSC-Exos reduced apoptosis of hepatocytes by inducing autophagy, evidenced by increased levels of autophagy marker proteins, microtubule-associated protein 1A/1B-light chain 3, Beclin-1, and the number of autophagosomes. Autophagy is a self-protection mechanism that attenuates liver cell death, functioning by removing damaged organelles and alleviating intracellular stress (Ni et al., 2012).

#### Kidney

Sepsis-associated acute kidney injury (S-AKI) is of high morbidity in severely ill patients, with high risk of developing into chronic kidney diseases and death (Peerapornratana et al., 2019). Multiple studies have provided histological and laboratory tests evidence to validate the capability of MSC-Exos in rescuing renal function in sepsis (Gang et al., 2021). In cecal ligation and puncture (CLP)-induced sepsis mice, the kidney morphology was more intact after intervention of MSC-Exos. Decreased kidney interstitial edema, higher integrity of brush borders and reduced inflammatory cell infiltration were observed in HE staining kidney tissues (Blanco et al., 2020; Gao et al., 2020). Blood tests further confirmed renal function restoration. In serum, the levels of blood nitrogen urea, serum creatinine and various inflammation indicators were downregulated after the treatment of MSC-Exos, indicating increased glomerular filtration rates (Nassar et al., 2016; Li et al., 2020b). The protective function is associated with the upregulation of SIRT1, which regulates NF-κB and apoptotic pathway (Gao et al., 2020). In another research, Shen et al. (2021) reported ADSC-Exos inhibited ROS accumulation and M1 polarization by downregulating Kelch Like ECH Associated Protein 1 and activating Transcription factor nuclear factor-E2-related factor 2 (Nrf2)/Heme Oxygenase-1 (HO-1) pathway (Shen et al., 2021). Other molecular mechanisms involve upregulation of miR-146b level in kidney tissue by hucMSC-Exos, which targets IL-1 receptor-associated kinase (IRAK1) and inhibits NF-κB activity (Zhang et al., 2020a).

#### Cardiovascular System

**Figure 5:**
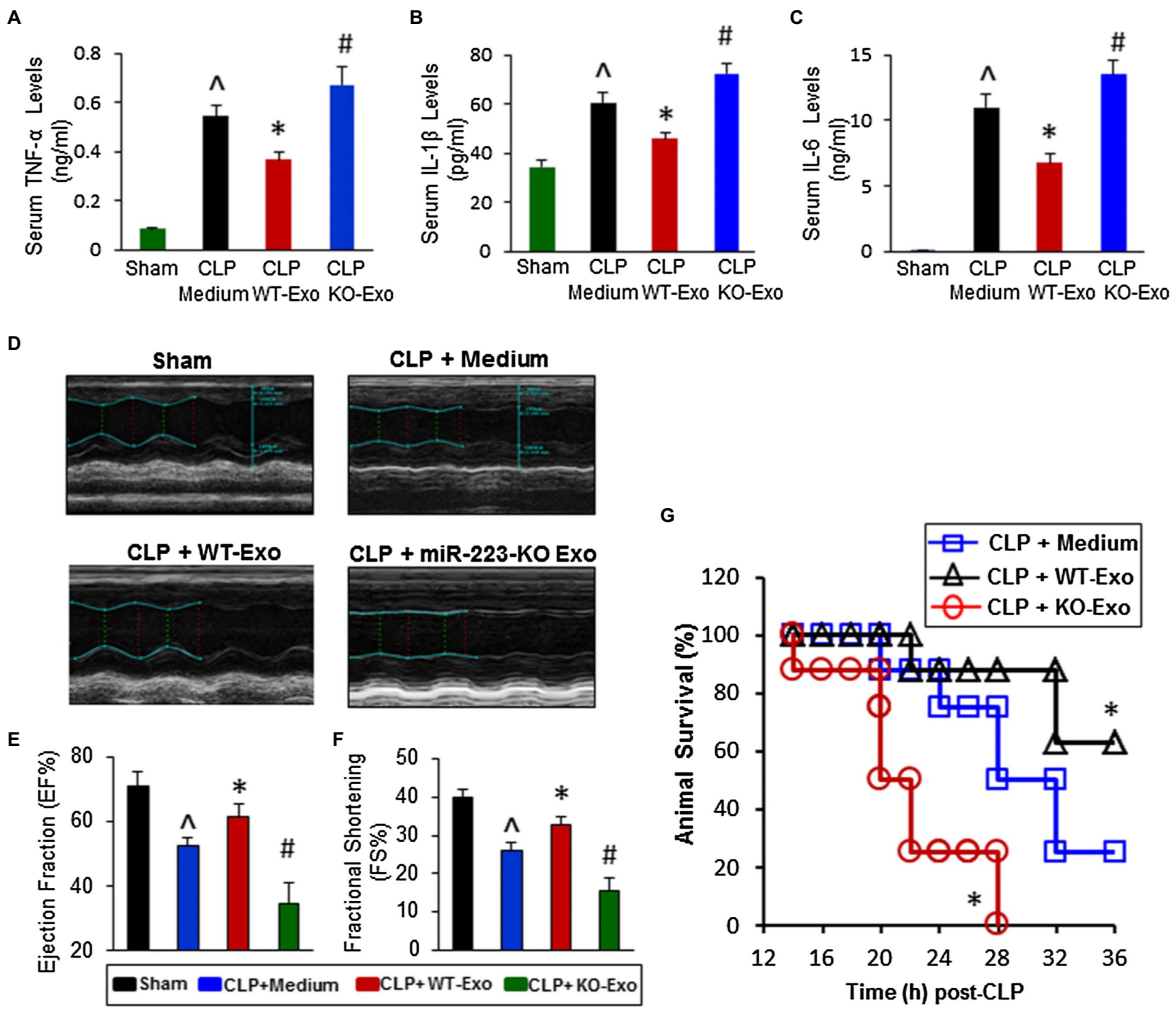
The effects of WT-exosomes and miR-223-KO exosomes on cecal ligation and puncture (CLP)-induced inflammatory response, cardiac dysfunction and animal mortality. **(A–C)** CLP-mice treated with WT-exosomes (*n* = 11) showed lower levels of serum TNF-α **(A)**, IL-1β **(B)**, and IL-6 **(C)**, whereas CLP-mice injected with KO-exosomes (*n* = 11) exhibited higher levels of circulating TNF-α **(A)**, IL-1β **(B)**, and IL-6 **(C)**, compared with those treated with incomplete DMEM medium (*n* = 10; ^^^*p* < 0.05 vs. shams; ^*^p < 0.05 vs. CLP + medium; ^#^*p* < 0.05 vs. CLP + medium). **(D)** Results of echocardiography measurement showed that values of the left ventricular ejection fraction (EF%, **E**) and the fractional shortening (FS%, **F**) were significantly decreased in CLP mice injected with incomplete DMEM medium (*n* = 10), compared with shams (*n* = 8). Remarkably, the reduction of EF% and FS% was attenuated in WT-exosome-treated CLP mice (*n* = 11); whereas it was aggravated in CLP mice administrated with miR-223-KO exosomes (*n* = 11; ^^^*p* < 0.05 vs. shams; ^*^p < 0.05 vs. CLP + medium; ^#^*p* < 0.05 vs. CLP + medium). **(G)** The survival of CLP-mice was significantly improved by WT-exosome treatment, whereas it was worse by miR-223-KO exosome injection (*n* = 8, ^*^p < 0.05 vs. CLP + medium; Adopted from (Wang et al., 2015) distributed under the creative commons CC BY license).

The pathogenesis of septic cardiomyopathy has not been fully revealed, and the current understanding of pathogenic mechanisms includes increased capillary permeability, oxidative stress, and calcium dyshomeostasis (Kakihana et al., 2016; Ehrman et al., 2018). MSC-Exos provide cardioprotection under septic conditions by suppressing inflammation and maintaining calcium homeostasis. Intravenous injection of MSC-Exos improved septic mice survival and inhibiting cardiomyocytes death by attenuating excess inflammation *via* miR-233, which downregulated Sema3A and Stat3 (Figure 5; Wang et al., 2015). Pink1 mRNA-containing hucMSC-Exos rescued injured cardiomyocytes by activating PINK1-PKA-NCLX axis, which alleviated cardiomyocyte mitochondrial Ca^2+^ efflux disorder (Zhou et al., 2021b). Anti-apoptotic effects on cardiomyocytes may associate with miR-21a-5p (Luther et al., 2018), miR-19a (Yu et al., 2015), miR-451 (Zhang et al., 2010), and miR-211 (Yu et al., 2013).

Taken together, MSC-Exos may represent a promising novel and efficacious cell-free therapeutic modalities in treatment of sepsis, depending on its orchestrated ability in anti-inflammation and anti-apoptosis.

### MSC-Exo Therapy for Bacteria-Associated Diabetic Foot Ulcers

Diabetic foot ulcers, characterized as micro-vascular dysfunction and peripheral neuropathy, is highly susceptible to secondary infections, as impaired immune function slows down wound healing and suppressed proprioception potentially leads to injuries. The biofilm on the wound site, produced by aerobic and anaerobic bacteria, exhibits resistance toward antibiotics that causes difficulties to the treatment of DFU (Raghav et al., 2021). Prevalence of polymicrobial or multidrug-resistant bacteria infections increased the difficulties to control infections, and the risk of amputation (Pitocco et al., 2019). In this part, we place particular emphasis on the mechanisms of action by which MSC-Exos confer the ability to accelerate wound healing and strengthen biological barriers against microbes in treating bacteria-associated DFU.

Mesenchymal stem cell-derived exosomes rescue function of epithelial progenitor cells (EPC) and promote angiogenesis by delivering RNAs (microRNAs, lncRNAs, and circRNAs) and proteins (Dalirfardouei et al., 2021). Several studies demonstrated hydrogels combined with MSC-Exos promoted wound healing and skin regeneration under diabetes conditions *via* the transfer of RNAs and proteins (Wang et al., 2019a,b). MiR-126 in exosomes from deferoxamine-pretreated BMSCs activated PI3K/AKT signaling pathway to promote angiogenesis in diabetic rat wounds (Ding et al., 2019). Synovial MSCs overexpressing miR-126 accelerated angiogenesis, re-epithelization, and maturation of collagen (Tao et al., 2017). ADSC-Exos overexpressing linc00511 enhanced proliferation, migration, and angiogenesis of EPC, by inhibiting Progestin and adipoQ receptor family member III (PAQR3) expression, and increasing Twist homolog 1 protein level by reducing its degradation (Qiu et al., 2021). ADSC-Exos were found to suppress apoptosis of EPC induced by high glucose through stimulating autophagy. The transmit of mmu_circ_0000250 inhibited miR-128-3p and upregulated expression of SIRT1, which promoted autophagy in EPCs (Shi et al., 2020). Protein cargoes also contribute to the improvement of EPC function. ADSC-Exos overexpressing Nrf2 alleviated senescence and oxidative stress of EPC under high glucose conditions, evidenced by improved levels of Senescence Marker Protein 30, and decreased levels of oxidative stress-related proteins (NADPH oxidase 1, NADPH oxidase 4; Li et al., 2018). Deleted in malignant brain tumors 1 (DMBT1), a pro-angiogenic protein, from human urine-derived stem cells (USCs) accounts for the pro-angiogenic effects of USC-Exos (Chen et al., 2018). Other activated signaling pathways include MAPK (Li et al., 2016) and NF-kB (Dalirfardouei et al., 2019) pathways.

The wound healing of DFU requires the collaboration of multiple types of cells. MSC-Exos also target fibroblasts and keratinocytes for the acceleration of re-epithelialization, collagen deposition, and remodeling *via* regulating MAPK/ERK (Li et al., 2016), PI3K/AKT (Li et al., 2015; Zhang et al., 2018; Sears et al., 2021), and Wnt/β-catenin (Lv et al., 2020) pathways. Engineered hADSC-Exos overexpressing miR-21 significantly strengthened the migration and proliferation of keratinocytes by upregulating Matrix Metallopeptidase 7 (Lv et al., 2020). Modified MSC-Exos transferred lncRNA H19 to fibroblast, which impaired miR-152-3p-mediated phosphatase and tensin homolog (PTEN) inhibition and thus suppressed PI3K/AKT pathway, leading to increased migration, proliferation, and decreased apoptosis of fibroblast (Li et al., 2020a).

Mesenchymal stem cell-derived exosomes also benefit the treatment of DFU by promoting neural repair to regain peripheral sensation. Shi et al. (2017) reported the combination of exosomes from GMSCs and chitosan/silk hydrogel increased nerve density, compared with the hydrogel group, indicating that GMSC-Exos might facilitate neuronal ingrowth into the wound bed (Shi et al., 2017). MiR-146a-overexpressing MSC-Exos constructed by Fan et al. (2021) promoted axon remyelination, and improved intraepidermal nerve fiber density in hind paw plantar skin of diabetic mice. In a word, MSC-Exos prevent infections of DFU by restoring blood supply, epidermis structure, and peripheral neuropathy.

## Clinical Translation of Exosome Therapy

### Clinical Trials

Clinical trials using stem cell-derived exosomes as intervention were searched by using the terms “exosomes” or “extracellular vesicles” in the ClinicalTrials.gov database,^1^ the European Union Clinical Trials Register^2^ and the World Health Organization International Clinical Trials Registry Platform (Chinese Clinical Trial Registry).^3^ Then selected studies went through manual screening to sort out microbial disease-related clinical trials. Eventually, 13 interventional studies and 2 expanded access are included, with observational studies excluded (Table 1). These clinical studies mainly aim at investigating the safety and efficacy of MSC-Exos in the treatment of infections. Some try to explore optimal dosage of exosomes by setting up different dosage intervention groups.

**Table 1:** Published clinical trials of mesenchymal stem cell-derived exosome (MSC-Exo) therapy in microbial diseases.

Trial ID number	Target disease	Stage	Study design	Sample volume	Exosome source	Route	Frequency
NCT04270006	Periodontitis	Early phase 1	Single group, open label	10	Adipose-derived stem cells	Location injection	-
ChiCTR1900027140	Chronic Periodontitis	N/A	Randomized parallel controlled study	48	Autologous dental pulp stem cells	Loaded on scaffold	-
NCT04602442	Covid19 pneumonia	Phase 2	Randomized, parallel, and double-blinded	90	Mesenchymal stem cells	Aerosol inhalation	Five times (every 2 days)
NCT04491240	Covid19 pneumonia	Phase 1, Phase 2	Randomized, parallel, and double-blinded	30	Mesenchymal stem cells	Aerosol inhalation	Five times (every 2 days)
NCT04657406	Mild to moderate COVID-19	Treatment IND	Expanded access	-	Amniotic stem cells and epithelial cells	Intravenous administration	Three times at day 0, 4, and 8
NCT04384445	Moderate to severe COVID-19	Phase 1, Phase 2	Controlled, randomized, parallel, and double-blinded	20	Amniotic stem cells and epithelial cells	Intravenous administration	Three times at day 0, 4, and 8
NCT04493242	Moderate-to-severe ARDS in patients with severe COVID-19	Phase 2	Multi-center, double-blinded, placebo-controlled, and randomized controlled	120	Bone marrow mesenchymal stem cells	Intravenous administration	-
NCT04276987	Covid19 pneumonia	Phase 1	Single group, open label	24	Allogenic adipose mesenchymal stem cells	Aerosol inhalation	Five times (each day)
NCT04657458	COVID-19 associated moderate to severe ARDS	Intermediate-size population	Expanded access	-	Bone marrow mesenchymal stem cells	Intravenous administration	-
NCT04798716	COVID-19 with moderate to severe NCP or ARDS	Phase 1, Phase 2	Open-label for the first 15 patients; RCT for the final 40 patients	55	Mesenchymal stem cells	Intravenous administration	Three times (every other day)
ChiCTR2000030484	Lung Injury following novel coronavirus pneumonia	N/A	Randomized parallel controlled study	90	Human umbilical cord mesenchymal stem cells	Intravenous administration	14 times (every day)
ChiCTR2000030261	Novel coronavirus pneumonia (COVID-19)	N/A	Randomized parallel controlled study	26	Mesenchymal stem cells	Aerosol inhalation	-
2021-002184-22	COVID-19 disease	Phase 2	Controlled, randomized, and single blinded	90	-	Aerosol inhalation	-
NCT04544215	Drug-resistant pulmonary infection	Phase 1, Phase 2	controlled, randomized, parallel, and double-blinded	60	Allogeneic human adipose-derived mesenchymal progenitor cells	Aerosol inhalation	Seven times (every day)
NCT04850469/ChiCTR2100044280	Sepsis (in children)	-	-	200	Mesenchymal stem cells	-	-

Most studies investigate systematic diseases, and only two of 15 studies (NCT04270006, ChiCTR1900027140) focus on a topical disease, periodontitis. Noteworthily, those two studies applied different routes of administration: One (NCT04270006) locally injects autogenous adipose stem cell exosomes into the periodontal pockets; while in another study (ChiCTR1900027140), the mixtures of DPSC-Exos, DPSCs, and Bio-Oss bone meal are applied during guided tissue regeneration. Changes in bone defect depth, pocket depth, clinical attachment loss, and gingival inflammation are measured to evaluate the degree of periodontal tissue regeneration in both studies. However, the lack of control group or blinding method may increase bias and compromise the reliability of outcomes.

The majority of trials (11 out of 15) are registered to investigate MSC-Exo treatment in COVID-19-associated pneumonia (one trial for Phase I, four for Phase I/II, two for Phase II, two for expanded access, and two for unclear phase). And one study (Phase I/II) focuses on drug-resistant pneumonia. Most studies are controlled, randomized, parallel, double-blinded trials, in which only NCT04493242 is conducted in multi-center. The parent cells sources are diverse, including bone marrow, adipose tissue, umbilical cord, amniotic fluid. Administration routes are roughly evenly split between intravenous injection and aerosol inhalation. Among 12 registered studies, only three (NCT04491240, NCT04493242, and NCT04276987) are completed. Results information of NCT04276987 is currently not publicly available. In NCT04491240, no adverse event was reported during both exosome solution inhalation procedure and the whole trial, indicating the safety of intranasal administration of MSC-Exos. Regarding therapeutic efficacy, it seems that no data (time to clinical recovery, SpO2 concentration, C-reactive Protein, and Lactic Acid Dehydrogenase level in serum) indicated a significant difference between exosome treatment groups and placebo group. NCT04493242 has been published to report the safety and efficacy of allogeneic BMSC-Exos (ExoFlo™) for severe COVID-19 infections. No adverse events were observed 72 h after exosome therapy. After one course of treatment, PaO_2_/FiO_2_ (an oxygenation indicator), neutrophils and lymphocytes (CD3^+^, CD4^+^, and CD8^+^) counts had significant increases. Meanwhile, acute phase reactants (C-reactive protein, ferritin, and D-dimer) declined. The results indicated ExoFlo™ therapy’s capacity in restoring lung function and promoting protective immune response (Sengupta et al., 2020). Although the study demonstrated a promising future of MSC-Exo therapy in COVID-19 infections, doubts and uncertainties remained. The study was lack of crucial details in terms of exosome production, characterization, biological properties, and dosage. Furthermore, standards in evaluating the correlation between adverse events and exosome therapy were also questioned. More data are needed to allow proper assessment of the medical value and deeper exploration of the molecular mechanisms of ExoFlo™ (Lim et al., 2020). Only one not-yet-recruiting study (NCT04850469/ChiCTR2100044280) is to explore the administration of MSC-Exos in sepsis. And it is also the only study that chooses children as test subjects, with the rest of studies only involving adult participants.

### Routes of Administration

Biodistributions and biological properties of exosomes vary depending on the application form. Therefore, it is of great importance to figure out optimal ways to deliver exosomes based on disease characteristics. Generally, there are two strategies for exosome administration, systematic administration, and topical administration. Systematic administration involves intravenous injection and aerosol inhalation, appropriate for multi-system diseases or internal organs-affected diseases, sepsis and novel corona pneumonia as typical examples. Conversely, topical administration is suitable for limited infections, with the hope that exosomes are constraint in the focus of infections and exert maximum curative effects to local cells.

#### Systematic Administration

##### Intravenous Administration

Intravenous administration might be the most common exosome delivery method in basic research or clinical trials. Generally, biodistributions and pharmacokinetics of exosomes vary depending on parent cell sources and patients’ pathologic conditions. Grange et al. (2014) performed intravenous injection of DiI-labeled MSC-EVs in healthy mice, and detected the fluorescent signal from freshly dissected tissues after 5 and 24 h. It showed that fluorescence intensity peaked in liver, spleen, and lung successively. While in an acute kidney injury mouse model, the accumulation of exosomes in the kidney increased and extended (Grange et al., 2014). Choi et al. (2021) observed over 80% of HEK293T cell-derived exosomes were cleared out from the circulation in 1 h after intravenous injection, with most of the rest tentatively accumulating in liver and then translocated to the intestine from 8 h post-injection. While in sepsis mice model, clearance speed significantly slowed down, indicating that liver dysfunction in later stage of sepsis may delay biliary excretion of exosomes (Choi et al., 2021). After intravenous injection, exosomes showed a short half-life (several minutes) in the circulation of healthy animals, most of which were captured by peripheral macrophages and neutrophils. Later, the remaining exosomes mostly accumulated in liver and spleen for more than 24 h. Rapid clearance from blood imposes restriction on the proportion of exosomes arriving target tissue, thus lessening therapeutic effects (Morishita et al., 2017; Yang et al., 2021).

##### Intranasal Administration

Intranasal exosome delivery refers to the transformation of exosome solution into aerosol which is inhaled directly into the lung (Pires et al., 2009). In this way, exosomes target lung tissue first, and go through lung air-blood barrier to target remote organs. It attracts increasing attentions in the treatment of COVID-19 associated pneumonia (Tsuchiya et al., 2020). Exosome nebulization results in a more homogeneous spread with deeper penetration to the distal lung lobules. Furthermore, it is non-invasive, almost painless, and convenient to conduct without the need for sterilization. More importantly, intranasal delivery may improve on-target effect in central neural system, as it overcomes difficulties in crossing blood-brain barrier by passing through neuroepithelium in nasal olfactory region to get direct access to the brain (Haney et al., 2015; Guo et al., 2019). Guo et al. (2019) labeled exosomes with glucose-coated gold nanoparticles (GNP), and tracked them by *in vivo* neuroimaging, giving a hint about the difference of intravenous and intranasal administration in brain accumulation and whole-body biodistribution. It turns out that the latter one made it easier to pass blood-brain barrier, leading to superior brain targeting, while the former one resulted in higher accumulation within the liver (Guo et al., 2019). However, consideration should be taken that pathological conditions in airway may affect nasal mucociliary clearance and influence drug absorption.

#### Topical Administration

Topical administration directs exosomes to sites of injection, suitable for superficial injuries or localized infections. For example, in open fractures, local administration of antibiotics leads to higher concentrations within the wound cavity, meanwhile minimizing systemic toxicity. In contrast, systemically administered antibiotics are hard to access avascular wound cavities (Lawing et al., 2015). The application forms are diverse, including local injection, smearing, exosome-loaded scaffolds, etc.

Gong et al. (2021) performed intramyocardial injection of gold nanoparticle-labeled exosomes in a myocardial infarction mouse model. *In vivo* CT imaging showed that majority of MSC-Exos remained in the MI area for up to 24 h after injection, and only few spread to other organs, indicating local injection as an effective way to deliver exosomes to limited treatment areas (Gong et al., 2021). Mohammed et al. (2018) studied the use of ADSC-Exos as adjunctive therapy to nonsurgical periodontal treatment. By local injection into periodontal pockets, the ADSC-Exo group revealed the best results with significantly higher area % of newly formed tissues in ligature-induced periodontitis rat model demonstrated by histologic study (Mohammed et al., 2018). Zhou et al. (2021c) compared two administration routes, smearing and subcutaneous injection, in the efficiency of delivering human ADSC-Exos to promote cutaneous wound healing. The results showed that the ADSC-Exo-smearing group significantly shortened healing time and narrowed scars on full-thickness wounds in mice. Specifically, HE staining and Masson staining illustrated better re-epithelialization and well-reorganized collagen fibers in the ADSC-Exo-smearing group than the subcutaneous injection group. The authors attributed this to the loss of exosomes during the local injection and direct injection may disturb the hierarchy of wound. Smearing might be an optional treatment options for clinical patients with exposed surface wounds accompanied with chronic infections (Zhou et al., 2021c).

The application of exosome-scaffold complexes is expected to repair tissue defects and release therapeutic exosomes at a controlled and sustained speed. Qian et al. (2020) created a multi-functioned chitosan-silk fibroin dressing with silver nanoparticles-loaded exosomes for infected wounds healing. The CTS-SF/SA/Ag-Exo dressing showed a sudden burst of exosome release at the beginning, and maintained release in low speed for up to 48 h, exerting constant antimicrobial and healing promotion effect. Shiekh et al. (2020) developed ADSC-Exo-embedded oxygen releasing cryogels as wound dressing, in which exosomes were released gradually for up to 6 days. The exosome-laden wound dressing improved the wound healing in *Staphylococcus aureus*, and *P. aeruginosa* infected diabetic wound ulcers, with enhanced collagen I deposition and mature epithelial structures observed (Shiekh et al., 2020). In other novel wound dressing, ADSC-Exos were encapsulated in the FHE hydrogel (F127/OHA-EPL) through electrostatic interaction and exhibited a representative long-term pH-responsive sustained release behavior. The exosome laden FHE hydrogel showed great potential in promoting chronic diabetic wound healing and complete skin regeneration (Wang et al., 2019a). Similarly, GMSC-derived exosome-chitosan/silk hydrogel sponge complex accelerates wound healing in a diabetic rat skin defect model (Shi et al., 2017). Hydrogel promoted better healing of rat bone defects with the addition of hucMSC-derived exosomes (Wang et al., 2020b).

### Exosome Modifications

Exosome modifications are an essential process to endow exosomes with more powerful therapeutic effects. Efforts are made with the purpose of increasing therapeutical components loadings and enhancing delivery efficiency.

#### Increasing Loading of Therapeutical Components

To improve the therapeutic capacity of exosomes, exosome modifications can be divided into pre-loading and post-loading two strategies, depending on the timing of intervention (de Jong et al., 2020).

##### Promoting Expressions of Bioactive Molecules

In the pre-loading approach, parent cells are pretreated with biochemical or biophysical stimulations. Endogenous bioactive molecules are then packaged into exosomes in the process of vesicle biogenesis. Alterations in extracellular environments change the synthesis patterns of proteins and nucleic acids in stem cells (Katsuda et al., 2013). Preconditioning with pro-inflammatory cytokines or virulence factors simulates the environment of early infections and tissue damages, in which MSCs adapt and prepare themselves to survive in harsh conditions, by releasing soluble factors or extracellular vesicles to regulate microenvironment and accelerate tissue repair (Munir et al., 2020). The activation of toll-like receptors (TLRs)/pathogen-associated molecular patterns (PAMPs) signaling pathway plays an important role in stimulating immune responses and tissue repair in MSCs (Shirjang et al., 2017). PAMPs, such as LPS (Ti et al., 2015), or synthetic ligands, such as Poly (I:C; Pierce and Kurata, 2021), can interact with TLRs in MSCs to initiate downstream pathways to enhance the antimicrobial and immunomodulatory proteomic profile of secreted EVs, which can promote M2 polarization and enhance pathogen phagocytosis of macrophages/monocytes. Similar enhancements in EVs’ biological properties can be acquired by preconditioning with pro-inflammatory cytokines, TNF-α (Nakao et al., 2021) and IFN-γ (Varkouhi et al., 2019). Apart from enhancing expressions of therapeutic substances, stimulations of pro-inflammatory cytokines or virulence factors also induce a larger amount of exosome secretion (Ti et al., 2015; Varkouhi et al., 2019; Nakao et al., 2021). Upregulation of specific microRNA expressions in MSCs endows exosomes with properties such as pro-angiogenesis (Li et al., 2016; Tao et al., 2017; Ding et al., 2019), immunomodulation (Fan et al., 2021), and anti-apoptosis (Yu et al., 2015; Shi et al., 2020). What is more, researchers have found that biophysical stimuli, such as hypoxia and ionizing radiation (Jabbari et al., 2019), can alter biomolecules composition of EVs to increase the pro-angiogenic property. Gorgun et al. (2021) reported inflammatory cytokines (TNF-α, IL-1α) and hypoxia exerted synergistic effects on improving the pro-angiogenic property of secreted MSC-EVs. Similarly, in another research, hypoxia-treated human ADSCs released exosomes with a greater pro-angiogenesis property in grafted tissue *via* regulating VEGF/VEGF-R signaling (Han et al., 2019).

##### Incorporation of Therapeutic Drugs

The post-loading approach refers to primitive exosome processing, in which therapeutic agents are internalized or attached to the isolated exosomes. Conventional post-loading methods include passive incubation, electroporation, sonication, and transfection (de Jong et al., 2020). Antimicrobial or immunomodulatory substances, such as antibiotics (Yang et al., 2021), antimicrobial nanoparticles (Qian et al., 2020), microRNA mimics (Lv et al., 2020), or other therapeutic drugs (Sun et al., 2010) can be loaded into exosomes. For instance, Yang et al. (2021) improved cell permeability of vancomycin, a hydrophilic antibiotic, by loading it into exosomes *via* sonication. With such modification, vancomycin was able to target and eradicate intracellular *Methicillin-resistant S. aureus*. Furthermore, exosomes as vectors helped the accumulation of antibiotics in liver and spleen, where infected macrophages were predominantly distributed. Encapsulating antibiotics with exosomes contributes to alleviating toxicity and possibility of antibiotic resistance by lowering the dosage of antibiotics (Yang et al., 2021). In another research, Qian et al. (2020) observed synergistic effects between exosomes and silver nanoparticles (AgNPs) in repairing infected wounds. The combination of exosomes and AgNPs formed a protein corona around the nanoparticles, which stabilized the nanoparticles and prevented agglomeration. When AgNP-Exos were administered in the infection site, AgNPs and bioactive molecules of exosomes were released by the lysis of exosome membranes *via* the phospholipase secreted by *P. aeruginosa*. AgNPs exhibited antimicrobial activity by interrupting the integrity of bacterial cell walls, meanwhile exosomes promoted epithelial, vascular, and nerve fiber regeneration (Qian et al., 2020). Analogously, the incorporation of curcumin into exosomes greatly improved the solubility, stability, and bioavailability of curcumin, which is an insoluble, hydrophobic polyphenol compound. Moreover, exosomes increased delivery of curcumin to activated monocytes because of target specificity. In an LPS-induced septic shock mouse model, exosomal curcumin group dramatically surpassed curcumin group in terms of downregulating the CD11b^+^Gr-1^+^ cell population, which was characteristics in acute lung inflammation (Sun et al., 2010). Generally, exosomes as drug delivery system help improve the solubility, stability, and bioavailability of therapeutic drugs. Moreover, the drug-exosome combination also benefits from the target specificity of exosomes.

#### Increasing Delivery Efficiency

There are two strategies in enhancing the on-target effect, minimizing sequestration by MPS and increasing tissue target specificity, which can be summarized as “eat me/do not eat me” strategy.

Exosomes are mainly cleared by MPS, which attributes to the short half-life of exosomes in blood circulation (Morishita et al., 2015). Camouflaging exosomes with anti-phagocytotic molecules is a feasible strategy to avoid MPS uptake, and thus extend exosomes’ half-life in circulation. Anti-phagocytotic candidate molecules that can be inserted or expressed on the surface of exosomes include CD47, CD24, CD44, CD31, β2M, PD-L1, App1, and DHMEQ (Parada et al., 2021). The time EVs stayed in the plasma doubled after CD47 modification, and improved biodistribution in targeted tissue was observed (Wei et al., 2021). When EVs were recognized by macrophages, the activation of CD47-SIRPα pathway initiated immune evasion and reduced the phagocytosis of EVs (Chao et al., 2012). Some evidence indicated clathrin heavy chain (Cltc) plays an important role in mediating endocytosis of exosomes in the liver and spleen. Wan et al. (2020) encapsulated siCltc into exosomes *via* electroporation to block endocytosis by mononuclear phagocyte system. *Via* such modification, exosomes’ biodistribution pattern was altered with less exosome detained in liver and spleen and more arriving target organs (Wan et al., 2020). In the treatment of lung cancer, Belhadj et al. (2020) developed a dosing scheme: First they saturated macrophage receptors with cationized mannan-decorated extracellular vesicles, and then injected chemotherapy drugs-loaded exosomes, which were functionalized with CD47, to further avoid sequestration in liver and target lung tissue. The combined strategy induced a 123.53% increase in tumor distribution compared to conventional nanocarriers (Belhadj et al., 2020).

Surface decorations that promote exosome-target cell interaction enhance precise delivery. Zhou et al. (2021a) strengthened the therapeutic effect of MSC-EVs in bacteria-associated ALI, by co-incubation of MSC-EVs with high molecular weight hyaluronic acid (HMW HA; 1.0 MDa). It seems that HMW HA played a role as the connecter between target cells and MSC-EVs, thus promoting trafficking, adhesion, and internalization of EVs. This process was mediated by the interaction between HA and CD44, a surface receptor enriched in both MSC-EVs and immune cells. Such modification boosted the therapeutic potency of EVs in *P. aeruginosa* pneumonia (Zhou et al., 2021a).

## Future Perspectives

Considering the breadth of research into exosome therapies, as well as the vast need for new therapeutic modalities for infectious diseases, this area of medicine is a growing field. Based upon these studies, we suggest that a combination of both host-directed and pathogen-directed therapeutic approaches may represent a valuable and exploitable strategy, over single therapies, to (i) control multidrug-resistant infections, (ii) minimize the risk of emergence of drug resistance, and (iii) reduce the time of therapy. There are many new approaches to improving the efficacy of exosome therapies, such as enhancing the on-target effect of exosome therapies or combining exosomes with existing drugs for synergistic effects. There are also many new research developments that will expand the possibilities for exosome therapies.

Although several clinical trials have preliminarily demonstrated the safety and efficacy of MSC-Exos in patients, because of cell source difference, the heterogeneity of MSCs is in the way of exosome quality control and comprehensive evaluation of different studies. For example, the consistency and uniformity of MSC-Exo quality cannot be quantified or guaranteed, when MSCs are extracted from different fat donors. A rigorous quality control system of MSC-Exo production is critical to reduce batch-to-batch variation. Therefore, overcoming the heterogeneity of stem cells is one of the most pressing issues in the process of clinical translation of exosome therapy. To tackle these challenges, MSCs can also be generated from pluripotent stem cells (PSCs) to overcome many limitations of above MSC sources (Lian et al., 2010). These MSCs can be derived from the same parental PSC to avoid disadvantages of adult MSCs i.e., batch-to-batch variations in MSC quality, stem cell senescence, and limited proliferative potency (Lian et al., 2016). Most recently, GMP-grade MSCs derived from human induced PSCs have been used in refractory graft-vs.-host-disease (GVHD) in clinical trials (Bloor et al., 2020). Exosomes produced from PSC-MSCs may provide another putative therapeutic tool to overcome many limitations of adult MSC-Exos or EVs (Thakur et al., 2021). Future work should focus on establishing international standards in quantifying the quality and consistency of MSC-Exo therapies. Proper completion and data sharing of existing clinical trials are needed in convenience of comprehensive evaluation and further study. Furthermore, technological breakthrough in industrial mass production of clinical-grade MSC-Exos is a prerequisite for extensive clinical applications.

## Author Contributions

YL, SJ, XW, and CD are involved in manuscript writing, conceptualization, and figure drawing and data analysis. YW and DH supervised and reviewed the manuscript. All authors contributed to the article and approved the submitted version.

## Funding

This work was supported by Beijing Natural Science Foundation (7214305), National Natural Science Foundation of China (81871492), Ten-Thousand Talents Program (QNBJ2019-2), Key R&D Plan of Ningxia Hui Autonomous Region (2020BCG01001), and China National Postdoctoral Program for Innovative Talents (BX20200020).

## Conflict of Interest

The authors declare that the research was conducted in the absence of any commercial or financial relationships that could be construed as a potential conflict of interest.

## Publisher’s Note

All claims expressed in this article are solely those of the authors and do not necessarily represent those of their affiliated organizations, or those of the publisher, the editors and the reviewers. Any product that may be evaluated in this article, or claim that may be made by its manufacturer, is not guaranteed or endorsed by the publisher.

## Footnotes

^1^ https://clinicaltrials.gov

^2^ https://www.clinicaltrialsregister.eu

^3^ http://www.chictr.org.cn
